# Development and validation of a nomogram for predicting moderate-to-severe complications following primary tumor resection in metastatic colorectal cancer

**DOI:** 10.3389/fonc.2026.1745535

**Published:** 2026-03-31

**Authors:** Yu Zou, Junjie Xiong, Kemei Zhong, Mingjun Yang, Yunlong Zhang, Jianbo Zhu, Jiaze Li, Kun Qian, Hui Li

**Affiliations:** Department of Gastrointestinal Surgery, The First Affiliated Hospital of Chongqing Medical University, Chongqing, China

**Keywords:** complications, metastatic colorectal cancer, nomogram, primary tumor resection, risk factors

## Abstract

**Background:**

Colorectal cancer has high incidence and mortality. Surgery is the primary curative treatment, but postoperative complications remain common. This study developed and validated a nomogram to predict moderate-to-severe complications after primary tumor resection(PTR) in metastatic colorectal cancer (mCRC).

**Method:**

A retrospective analysis of clinical data was conducted for mCRC patients undergoing PTR at our institution between January 2022 and December 2024. All patients were randomly divided into two groups: 70% for development and 30% for validation. Univariate and multivariate logistic regression analyses were conducted to identify the independent risk factors associated with moderate-to-severe complications occurring within 30 days postoperatively. Correlation heatmaps and Lasso regression analysis were employed to systematically screen and identify the most relevant variables. Subsequently, a nomogram was developed based on the significant predictors. The area under the curve (AUC) was determined based on the receiver operating characteristic (ROC) curve for assessing the predictive probability. A calibration curve was generated to contrast the predicted probability against the observed probability. The clinical utility of the nomogram was evaluated using decision curve analysis (DCA). Internal 10-fold cross-validation was performed using bootstrapping, and boxplots as well as the average calibration curve were generated to visualize the results.

**Results:**

A total of 404 mCRC patients receiving PTR treatment were enrolled, including 282 in the development group and 122 in the validation group. Of these, 32% (90) in the development group and 39% (47) in the validation group experienced moderate-to-severe postoperative complications. Multivariate Logistic regression analysis identified age (p = 0.017, OR = 1.041, 95% CI: 1.007–1.076), preoperative albumin level (p < 0.001, OR = 0.774, 95% CI: 0.704–0.851), tumor location (p = 0.012, OR = 2.243, 95% CI: 1.216–4.906), and operation duration (p < 0.001, OR = 1.007, 95% CI: 1.003–1.011) as independent risk factors for moderate-to-severe complications after PTR surgery. Based on these findings, a nomogram was developed and validated.

**Conclusion:**

This study identified four independent risk factors for moderate-to-severe complications in mCRC patients after PTR surgery and developed a reliable predictive model to assist surgeons in optimizing perioperative management for high-risk cases.

## Introduction

Colorectal cancer (CRC) ranks as the third most prevalent malignant tumor globally and accounts for the second highest number of cancer-related deaths (1). The high incidence and mortality rates of colorectal cancer can be attributed to a range of complex factors, primarily the global population aging and the unhealthy lifestyle habits (2).Despite significant progress in imaging technology and the widespread promotion of screening programs, which have moderately improved early detection rates, a considerable number of patients continue to be diagnosed at intermediate or advanced stages due to ignored symptoms or lack of adherence to standardized screening, resulting in poor prognosis and high mortality (3, 4). Approximately 20-25% of CRC patients are diagnosed with synchronous metastasis, and an additional 30-50% eventually develop metachronous metastasis during the course of their disease (5). The management of metastatic colorectal cancer (mCRC) is characterized by its complexity and prolonged nature, often requiring a multimodal strategy. Despite significant advancements in treatment modalities, with the 5-year survival rate for stage IV disease estimated to be approximately 14% (6).

The advent of novel targeted therapies, immunotherapies, and advanced local treatment modalities has enabled an increasing proportion of initially unresectable mCRC patients to undergo radical resection with curative intent (7, 8). Even in cases where complete resection of metastatic lesions is not feasible, primary tumor resection (PTR) can enhance long-term survival rates while reducing the risk of local complications, including bleeding, obstruction, and perforation (9–11). According to research reports, more than 15% of mCRC patients ultimately require emergency surgery as a result of these complications. Such complications are linked to an elevated perioperative mortality rate (5-10%), a higher stoma formation rate (20-40%), and an increased conversion rate to open surgery (30-50%) (12, 13), emergency surgery itself also represents an independent risk factor for surgical safety (14).

The standardized application of laparoscopic techniques has significantly enhanced the safety and efficacy of radical resection for colorectal cancer. Compared with traditional open surgery, this approach offers advantages such as less trauma, faster postoperative recovery, and shorter hospital stays. Multiple high-quality clinical studies have also demonstrated that its oncological outcomes are not inferior to, and in some cases even superior to, those of open surgery (15–17). However, postoperative complications remain unavoidable. Studies have reported that the incidence of postoperative complications in mCRC patients following PTR ranges from 20-40%. These complications not only extend hospital stays and increase medical expenses, but also negatively impact long-term survival and quality of life (QoL) (18, 19).

The Clavien-Dindo classification system is widely used to categorize postoperative complications. Complications graded II or higher, which require active intervention with medication or surgical measures, are classified as moderate to severe (20, 21).

In view of the high incidence and substantial clinical consequences of postoperative complications, there is an urgent requirement for a robust predictive model to identify and stratify high-risk metastatic colorectal cancer (mCRC) patients undergoing resection of the primary tumor (PTR). This study aims to develop and validate a nomogram for predicting moderate-to-severe complications (Clavien-Dindo classification ≥ II) following PTR in mCRC patients.

## Materials and methods

### Patient selection

We retrospectively collected clinical data from patients with metastatic colorectal cancer (mCRC) who underwent PTR at a single teaching hospital between January 2022and December 2024. The inclusion criteria were as follows: 1) Patients diagnosed with colorectal cancer via preoperative pathology; 2) Patients confirmed to have metastatic colorectal cancer through imaging or surgical exploration; 3) Patients who underwent laparoscopic resection of primary tumors at our institution. The exclusion criteria were as follows: 1) Patients who had previously undergone radical colorectal cancer surgery; 2) Patients requiring emergency surgery due to complete intestinal obstruction, perforation, or massive bleeding; 3) Patients with multiple primary cancers or concurrent malignancies in other systems; 4) Patients whose laparoscopic procedures were converted to open surgery; 5) Patients who underwent Miles or Hartmann procedures were excluded from the analysis, as the intraoperative creation of a colostomy inherently precludes the occurrence of postoperative anastomotic complications, such as anastomotic leakage; 6) Patients with incomplete baseline or surgical information. Ultimately, 404 patients with complete data were included in the study and randomly divided into the development group (n = 282) and the validation group (n = 122) at a ratio of 7:3. This study was approved by the Ethics Committee of the First Affiliated Hospital of Chongqing Medical University (approval number: 2025-020-01).

### Treatment strategies for mCRC

mCRC refers to colorectal cancer with distant metastases, and its management remains a subject of considerable debate in the international oncology community. At our institution, for patients with initially resectable mCRC, synchronous resection of both the primary tumor and metastatic lesions is performed in a single surgical stage when complete removal is technically feasible. For those presenting with initially unresectable disease, conversion therapy is prioritized, utilizing chemotherapy in combination with targeted agents or immunotherapy to achieve tumor downstaging or volume reduction, thereby increasing the likelihood of achieving curative-intent resection. In cases where metastatic lesions cannot be resected simultaneously but the primary tumor causes complications such as partial bowel obstruction or hemorrhage, we perform upfront resection of the primary lesion, followed by adjuvant chemotherapy. Targeted and immunotherapies are integrated according to established clinical indications. The feasibility of a subsequent second-stage metastasectomy is then assessed through periodic imaging evaluations. This comprehensive treatment approach is designed to deliver individualized, curative-oriented therapeutic strategies that optimize outcomes for both patients and their families. At our institution, only approximately 20% of patients underwent synchronous one-stage resection.

### Data collection

We retrospectively collected the baseline characteristics and surgical details of the patients. The baseline characteristics included age, gender, Body mass index (BMI), smoking and drinking history, history of previous abdominal surgery (PAS), neoadjuvant therapy, extent of metastasis, preoperative comorbidities, preoperative albumin levels, hemoglobin levels, tumor location, depth of tumor invasion, and tumor size. The surgical details included operation duration, intraoperative blood loss, need for blood transfusion, and postoperative complications (Postoperative complications requiring prompt intervention: pneumonia, abdominal infection, intestinal obstruction, anastomotic leakage, anastomotic hemorrhage, anastomotic stenosis, and intra-abdominal hemorrhage). Tumor location was categorized into proximal colorectum (cecum, ascending colon, and transverse colon near the hepatic flexure) and distal colorectum (transverse colon near the splenic flexure, descending colon, sigmoid colon, and rectum). Preoperative comorbidities included hypertension, diabetes (DM), chronic cardiovascular and cerebrovascular diseases (CVD) and chronic pulmonary diseases (CPD).

### Statistical analysis

All data were processed and statistically analyzed using SPSS (version 27.0) and R (version 4.4.3). The Kolmogorov-Smirnov test was employed to evaluate the normality of continuous variables. Continuous variables following a normal distribution were presented as mean ± standard deviation (SD) and compared using the t-test; non-normally distributed continuous variables were expressed as median (interquartile range, IQR) and compared using the Mann-Whitney U test. Categorical variables were summarized as frequency (percentage) and analyzed using the chi-square test or Fisher’s exact test when appropriate. Research results were reported as odds ratio (OR), 95% confidence interval (CI) and corresponding P value. A three-stage variable selection process was implemented: univariate logistic regression analysis identified candidate variables (p < 0.05); collinearity of continuous variables was assessed using correlation heatmaps, and variables with a correlation coefficient > 0.6 were excluded (22); LASSO regression with 20-fold cross-validation further eliminated variables that did not significantly contribute to the model. Significant predictors retained after these steps underwent multivariate logistic regression analysis (p < 0.05). Finally, a nomogram was constructed based on the significant predictors identified by LASSO regression and multivariate logistic regression to predict the risk of moderate-to-severe complications following PTR surgery.

The predictive model was evaluated using the following approaches. First, the receiver operating characteristic curve (ROC) of the nomogram-predicted probability was calculated, and the area under the curve (AUC) was utilized as a metric to assess the model’s predictive accuracy. An AUC exceeding 0.7 suggests that the model demonstrates satisfactory predictive performance (23, 24). Second, calibration curves were constructed to evaluate the model’s calibration capability (25). Third, decision curve analysis (DCA) was performed to assess the clinical net benefit of the nomogram (25). Finally, to prevent overfitting due to simultaneous model development and validation within the same dataset, the model’s stability was examined via bootstrap-based 10-fold internal cross-validation. Box plots and average calibration curves were generated to visually illustrate the validation outcomes (26).

## Results

### The research process

**Figure 1:**
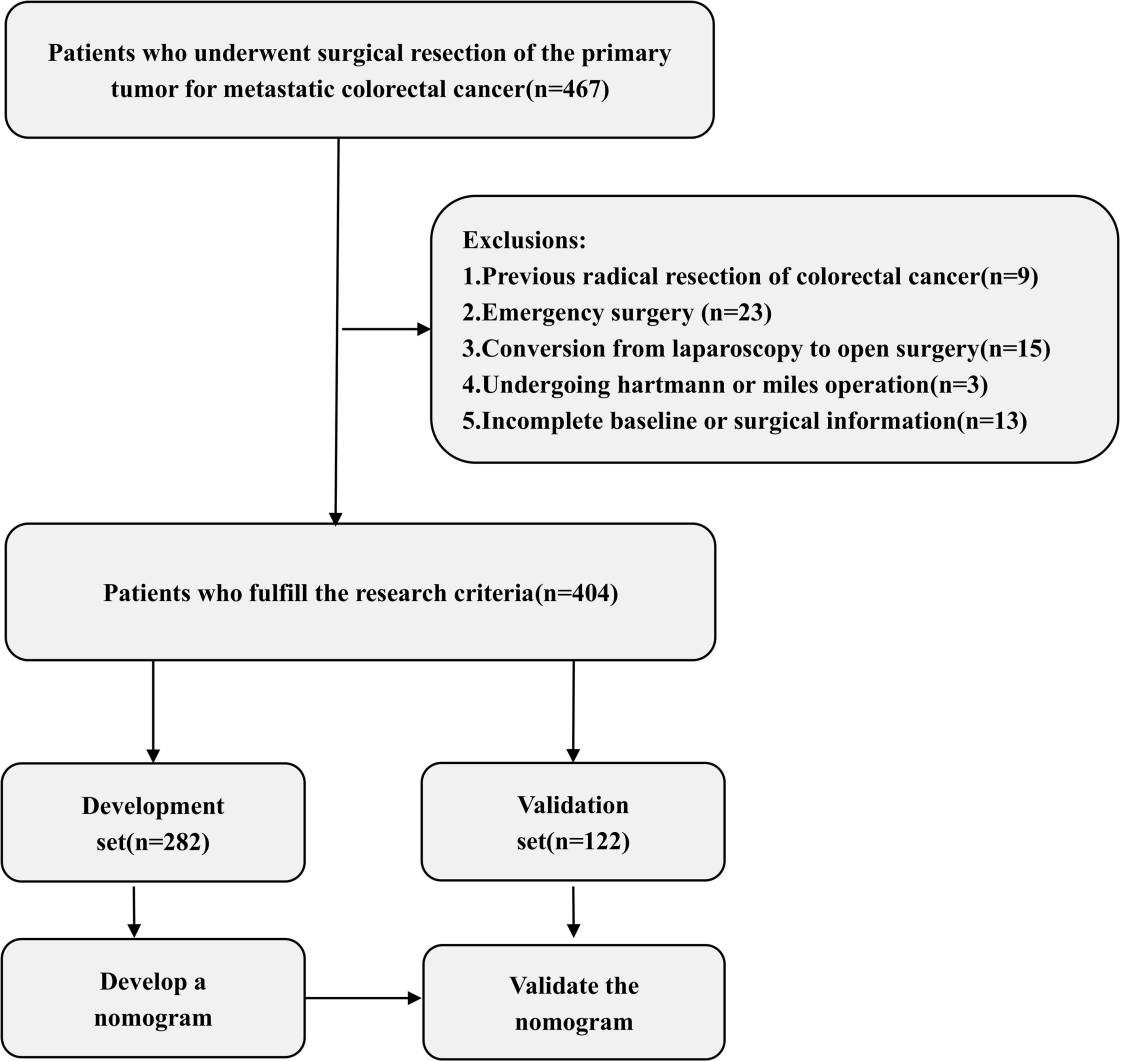
Flow chart of the study.

This study retrospectively analyzed 467 patients who underwent primary tumor resection at our hospital between January 2022 and December 2024. Among these patients, 63 were excluded due to a history of radical colorectal cancer surgery, emergency operations, conversion to open laparotomy, Hartmann or Miles procedures, or incomplete data (**Figure 1**). The remaining 404 patients were randomly allocated into the development group (282 patients) and the validation group (121 patients). Postoperatively, 137 patients experienced moderate-to-severe complications.

### Baseline data

According to the aforementioned inclusion and exclusion criteria, a total of 404 patients who underwent PTR surgery were enrolled in this study. These patients were randomly allocated into the development group (n = 282) and the validation group (n = 122). No statistically significant differences were observed in the baseline characteristics between the two groups (**Table 1**).

**Table 1:** Baseline information between the development and validation cohorts.

Characteristics	Total (n = 404)	Development (n = 282)	Validation (n = 122)	p
Complication				0.240
No	267 (66)	192 (68)	75 (61)	
Yes	137 (34)	90 (32)	47 (39)	
Age	63 (55, 70)	63 (55.25, 70)	63.5 (55.25, 69)	0.677
Sex				0.106
Female	148 (37)	111 (39)	37 (30)	
Male	256 (63)	171 (61)	85 (70)	
BMI	23.22 ± 3.25	23.09 ± 3.07	23.5 ± 3.63	0.277
Hb	119.5 (104, 132)	118.5 (104, 133)	120.5 (106.5, 130.75)	0.819
Alb	39 (36, 42)	39 (36, 42)	38 (36, 42)	0.539
Smoke				0.243
No	254 (63)	183 (65)	71 (58)	
Yes	150 (37)	99 (35)	51 (42)	
Drink				0.534
No	281 (70)	193 (68)	88 (72)	
Yes	123 (30)	89 (32)	34 (28)	
PAS				0.555
No	312 (77)	215 (76)	97 (80)	
Yes	92 (23)	67 (24)	25 (20)	
Hypertension				0.804
No	293 (73)	203 (72)	90 (74)	
Yes	111 (27)	79 (28)	32 (26)	
DM				0.697
No	352 (87)	244 (87)	108 (89)	
Yes	52 (13)	38 (13)	14 (11)	
CVD				0.598
No	375 (93)	260 (92)	115 (94)	
Yes	29 (7)	22 (8)	7 (6)	
CPD				1.000
No	389 (96)	271 (96)	118 (97)	
Yes	15 (4)	11 (4)	4 (3)	
Tumor location				0.414
Proximal colorectum	119 (29)	87 (31)	32 (26)	
Distal colorectum	285 (71)	195 (69)	90 (74)	
Combined organ resection				0.585
No	299 (74)	206 (73)	93 (76)	
Yes	105 (26)	76 (27)	29 (24)	
Operation duration	195 (150, 265)	195 (148.5, 265)	190 (160, 263.75)	0.520
Intraoperative bleeding	50 (50, 100)	50 (50, 100)	50 (30, 100)	0.072
T4 stage	333	228	105	0.262
Tumor size	3.7 (2.5, 5)	3.5 (2.5, 4.8)	4 (2.85, 5)	0.436
Blood transfusion				1.000
No	395 (98)	276 (98)	119 (98)	
Yes	9 (2)	6 (2)	3 (2)	
Neoadjuvant therapy				0.931
No	262 (65)	182 (65)	80 (66)	
Yes	142 (35)	100 (35)	42 (34)	
M stage				0.359
M1	314 (78)	222 (79)	92 (75)	
M2	41 (10)	30 (11)	11 (9)	
M3	49 (12)	30 (11)	19 (16)	
ASA				0.720
≤2	146 (36)	104 (37)	42 (34)	
>2	258 (64)	178 (63)	80 (66)	

### Screen the nomogram variables

**Figure 2:**
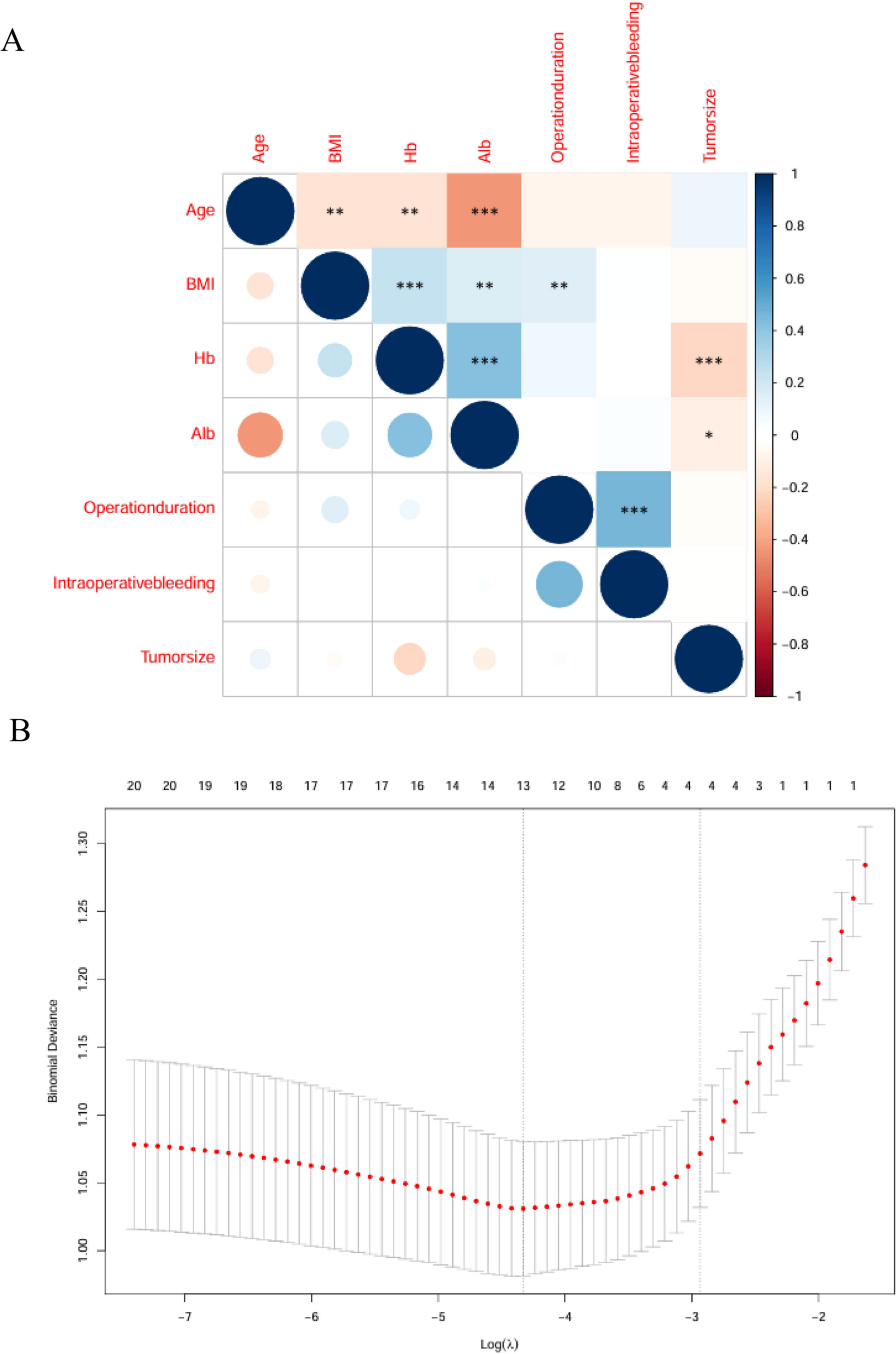
Correlation heat map **(A)**. Cross-validation diagram **(B)**.

**Figure 3:**
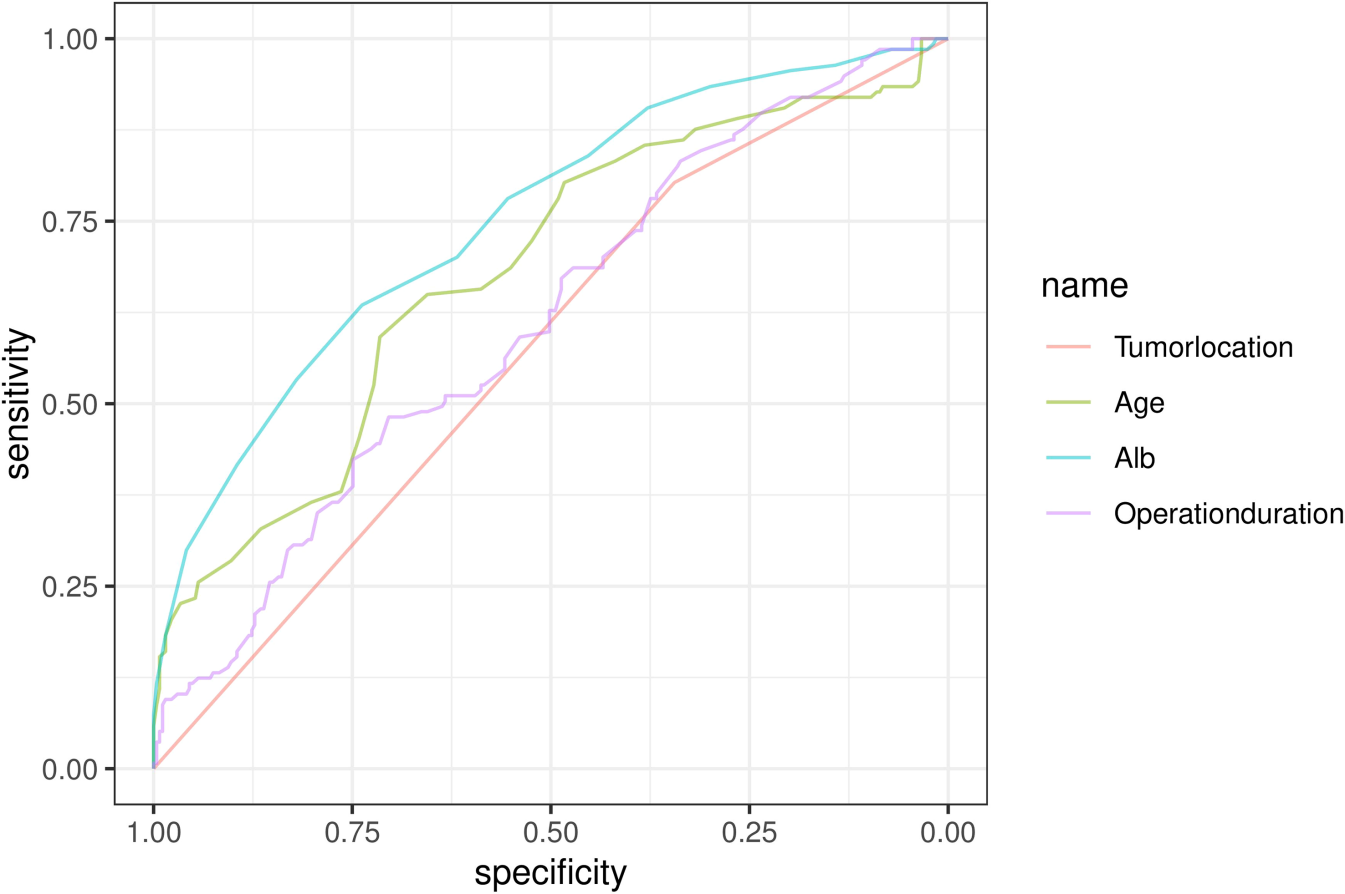
The ROC results for all independent risk factor.

Univariate and multivariate logistic regression analyses (including baseline characteristics and surgical information) were performed to identify risk factors associated with the incidence of moderate-to-severe complications after PTR. Univariate logistic regression analysis revealed that age (p < 0.001, OR = 1.068, 95% CI: 1.038 - 1.098), preoperative albumin level (p < 0.01, OR = 0.770, 95% CI: 0.709 - 0.835), tumor location (p = 0.033, OR = 1.876, 95% CI: 1.052 - 3.347), and operation duration (p < 0.001, OR = 1.006, 95% CI: 1.003 - 1.009) were potential risk factors for moderate-to-severe complications following PTR. Following collinearity analysis, a correlation heatmap and cross-validation plot were generated (**Figure 2**), demonstrating that all variables identified in the univariate analysis exhibited correlation coefficients below 0.6. Subsequently, multivariate logistic regression was conducted on the preoperative variables identified as significant in the univariate analysis. Multivariate logistic regression confirmed that age (p = 0.017, OR = 1.041, 95% CI: 1.007 - 1.076), preoperative albumin level (p < 0.001, OR = 0.774, 95% CI: 0.704 - 0.851), tumor location (p = 0.012, OR = 2.243, 95% CI: 1.216 - 4.906), and operation duration (p < 0.001, OR = 1.007, 95% CI: 1.003 - 1.011) were independent risk factors for moderate-to-severe complications after PTR (**Table 2**). The ROC curves for each independent risk factor are presented in **Figure 3**. The AUC values for these factors were calculated as follows: age (0.684), preoperative albumin level (0.751), tumor location (0.574), and operation duration (0.612). The Omnibus test of model coefficients yielded a χ2 value of 80.901 (P < 0.001), indicating that the logistic regression model was statistically significant. Additionally, the Hosmer-Lemeshow test produced a P value of 0.239, suggesting an adequate fit of the model to the observed data.

**Table 2:** Univariate and multivariate logistic regression analysis of complications (CD≥2).

Risk factor	Univariate Logistic regression analysis		Multivariate logistic regression analysis	
	OR (95%CI)	P	OR (95%CI)	P
Age	1.068 (1.038-1.098)	<0.001	1.041 (1.007-1.076)	0.017
Sex (female/male)	1.459 (0.865-2.464)	0.157		
BMI	1.004 (0.925-1.089)	0.932		
Hb	0.995 (0.983-1.006)	0.364		
Alb	0.77 (0.709-0.835)	<0.001	0.774 (0.704-0.851)	<0.001
Smoke (Yes/No)	1.186 (0.705-1.996)	0.520		
Drink (Yes/No)	0.899 (0.522-1.547)	0.700		
PAS (Yes/No)	0.881 (0.486-1.600)	0.678		
Hypertension (Yes/No)	1.249 (0.721-2.163)	0.428		
DM (Yes/No)	1.289 (0.632-2.630)	0.484		
CVD (Yes/No)	0.606 (0.216-1.697)	0.34		
CPD (Yes/No)	1.824 (0.5410-6.141)	0.332		
Tumor location (proximal/distal)	1.876 (1.052-3.347)	0.033	2.443 (1.216-4.906)	0.012
Combined organ resection (Yes/No)	1.716 (0.992-2.970)	0.053		
Operation duration	1.006 (1.003-1.009)	<0.001	1.007 (1.003-1.011)	<0.001
Intraoperative bleeding	1.001 (0.999-1.003)	0.159		
T4stage (Yes/No)	1.025 (0.542-1.940)	0.939		
Tumor size	1.028 (0.909-1.164)	0.657		
Blood transfusion (Yes/No)	2.172 (0.430-10.981)	0.348		
Neoadjuvant therapy (Yes/No)	1.080 (0.641-1.819)	0.772		
Mstage (1/2/3)		>0.050		
ASA (≤2/>2))	1.348 (0.795-2.288)	0.268		

PAS, history of previous abdominal surgery; BMI, Body mass index; DM, diabetes; CVD, chronic cardiovascular and cerebrovascular Diseases; CPD, chronic pulmonary diseases; ASA, American Society of Anesthesiologists.

### Development of a nomogram

**Figure 4:**
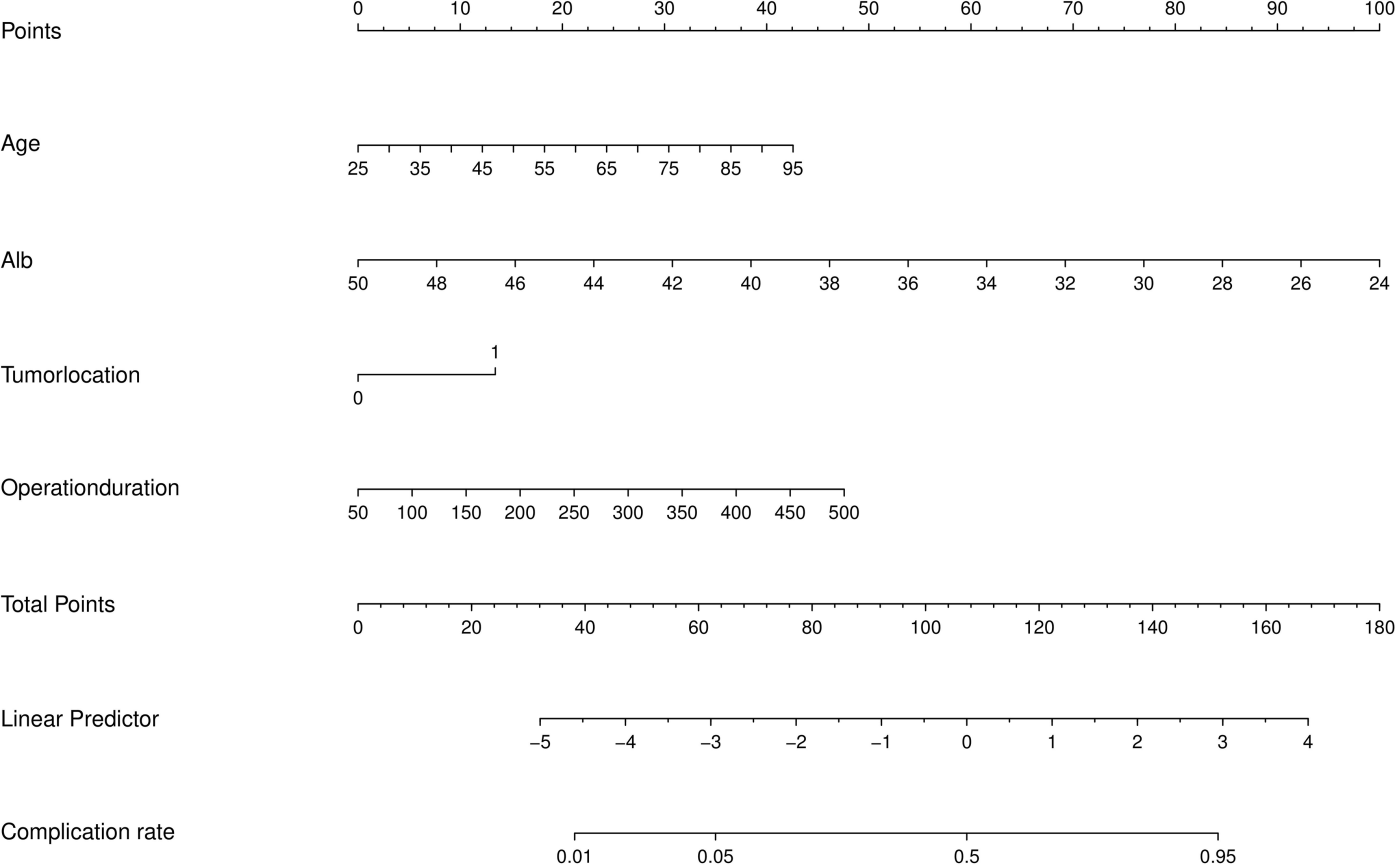
Nomogram for predicting moderate-to-severe complications following primary tumor resection(PTR) in metastatic colorectal cancer (mCRC). tumor location, proximal colorectum (0) and distal colorectum (1).

A nomogram model was developed using the four independent risk factors identified via multivariate logistic regression analysis to estimate the likelihood of moderate-to-severe postoperative complications in mCRC patients. As illustrated in **Figure 4**, each factor’s score is assigned based on the patient’s clinical profile, and the overall score is obtained by aggregating the scores of these four factors. The final predicted probability of moderate-to-severe postoperative complications corresponds to their total score.

### Validation of a nomogram

**Figure 5:**
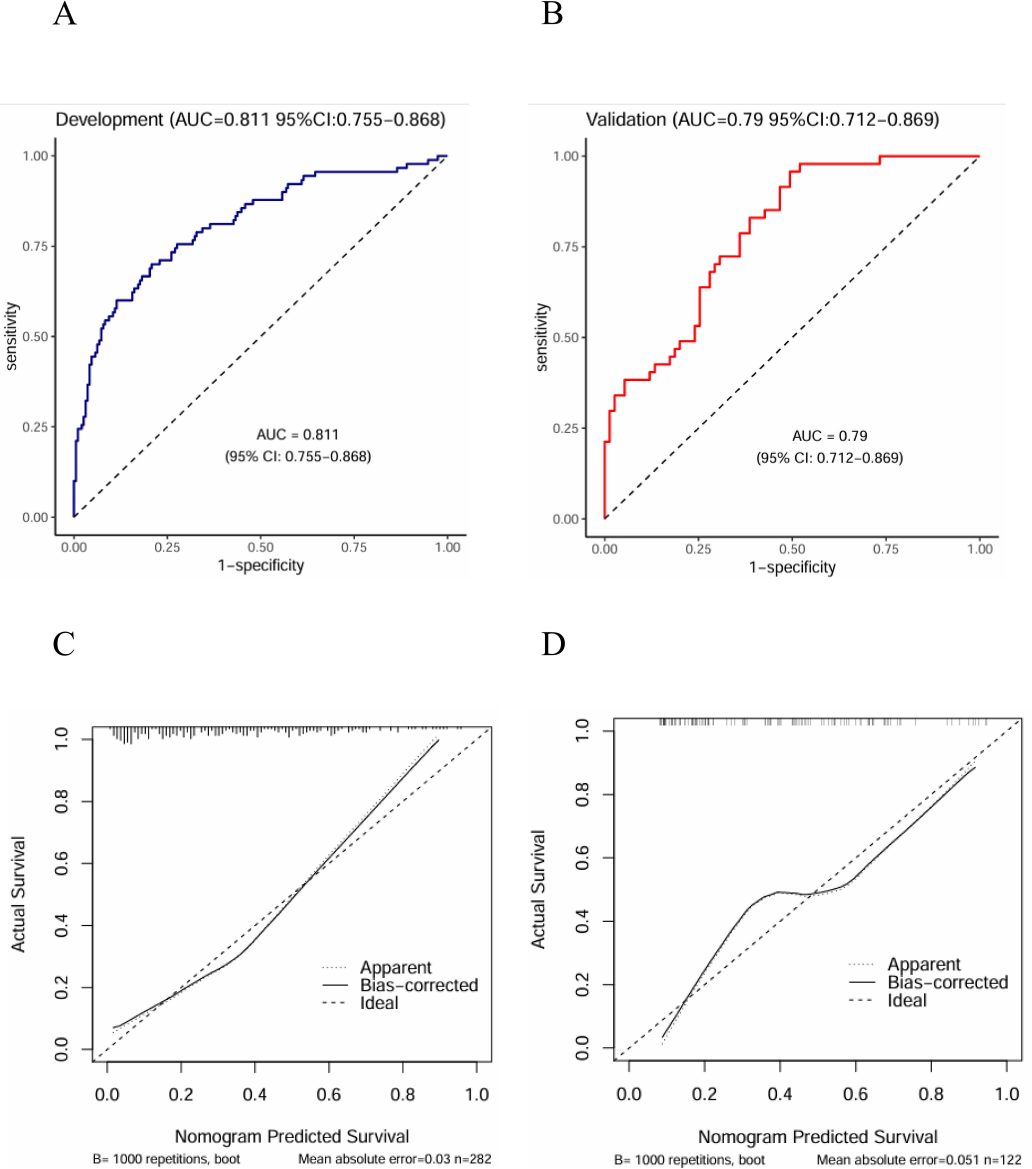
ROC of the development set **(A)** and the validation set **(B)**, calibration of the nomogram in the development set **(C)** and the validation set **(D)**.

**Figure 6:**
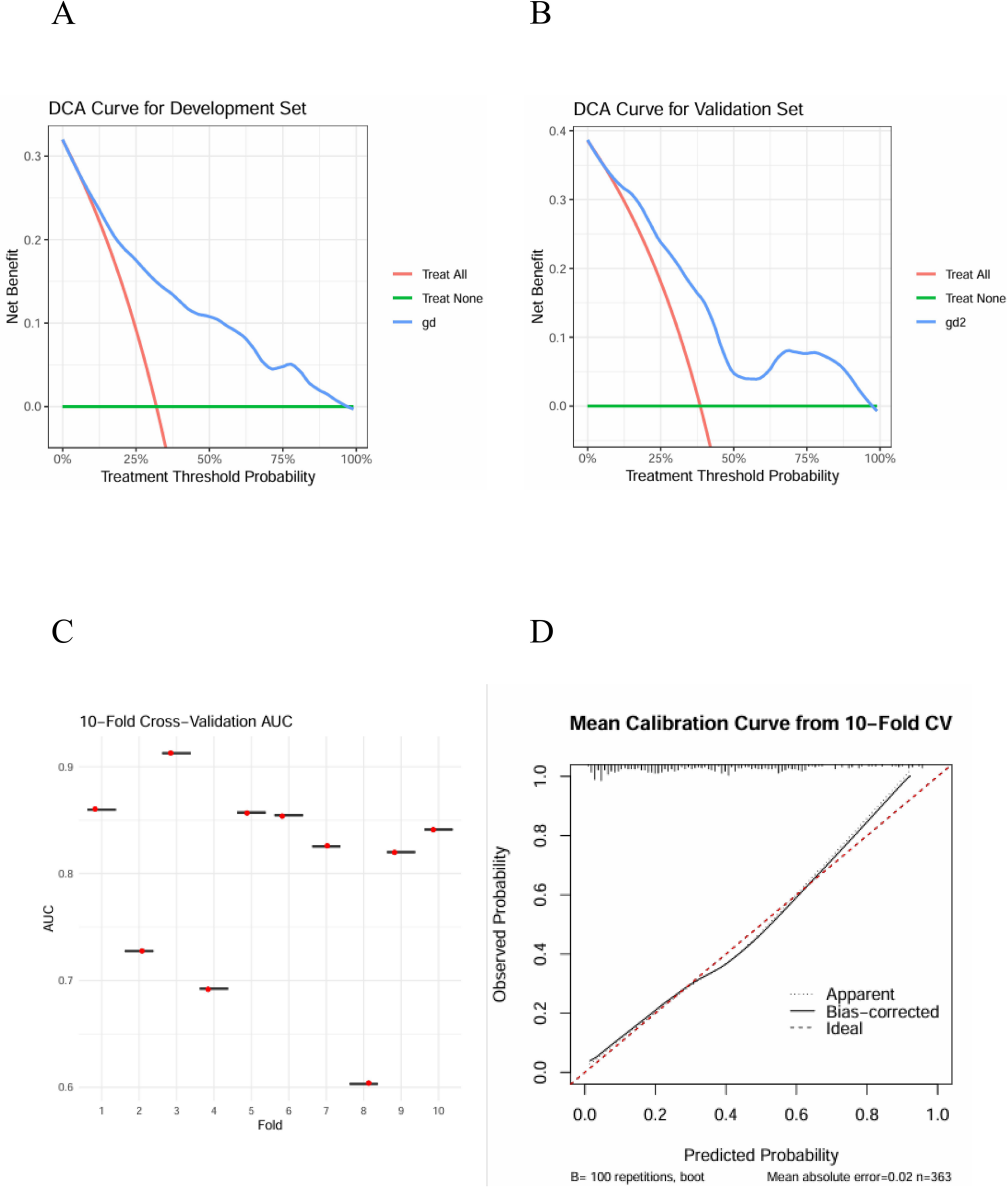
DCA Curve of development set **(A)** and the validation set **(B)**, ten-fold cross validation AUC **(C)**, the overall average AUC was (0.799 ± 0.0.095). 10-fold cross-validation average calibration curve **(D)**.

The ROC curve was utilized to assess the predictive accuracy of the nomogram. The results revealed that the AUC for the development group was 0.811 (95% CI: 0.755 - 0.868), while the AUC for the validation group was 0.790 (95% CI: 0.712 - 0.869) (**Figure 5**). The calibration curve (**Figure 5**) demonstrated that the prediction outcomes of the nomogram model developed in this study were closely aligned with the observed results. The DCA curve (**Figure 6**) confirmed that the model exhibited satisfactory clinical utility. The boxplot (**Figure 6**), which represented the average AUC value (0.799 ± 0.095), indicated that the model possessed robust stability. Furthermore, the average calibration curve (**Figure 6**) illustrated that the prediction results were highly consistent with the observed results, reflecting excellent predictive performance.

## Discussion

This study focused on developing and validating a nomogram designed to estimate the likelihood of moderate-to-severe complications after primary tumor resection (PTR) in patients with metastatic colorectal cancer (mCRC). Our retrospective cohort analysis involved 404 patients who had undergone PTR, with 282 patients allocated to the development group (70%) and 122 patients assigned to the validation group (30%). The incidence of moderate-to-severe postoperative complications was 32% in the development group and 39% in the validation group, aligning with the rates reported in prior studies. Multivariate logistic regression analysis identified age (p = 0.017, OR = 1.041, 95% CI: 1.007 - 1.076), preoperative albumin (p < 0.001, OR = 0.774, 95% CI: 0.704 - 0.851), tumor location (p = 0.012, OR = 2.243, 95% CI: 1.216 - 4.906), and operation duration (p < 0.001, OR = 1.007, 95% CI: 1.003 - 1.011) as independent risk factors for complications. A nomogram developed based on these predictors exhibited excellent predictive performance, achieving an AUC of 0.811 (95% CI: 0.755 - 0.868) in the development group and 0.790 (95% CI: 0.712 - 0.869) in the validation group. The calibration curve demonstrated a strong alignment between predicted and actual probabilities, reflecting high predictive accuracy of the nomogram. Decision curve analysis (DCA) validated the clinical utility of the nomogram. Results from 10-fold cross-validation, presented via boxplots, showed an average AUC of 0.799 ± 0.095, indicating robust model stability. Additionally, the average calibration curve confirmed a high degree of consistency between predicted and observed outcomes.

In 2009, surgeons from seven global centers reassessed the system and categorized complications of grade II or higher (requiring active medical or surgical intervention) as moderate-to-severe complications (27).

Previous studies have identified various risk factors for postoperative complications in patients with mCRC, such as age, albumin level, tumor location, and operation duration. In some earlier studies, male gender was recognized as an independent risk factor for postoperative complications in colorectal cancer patients, potentially due to a higher prevalence of smoking and drinking history among male patients, as well as their deeper and narrower pelvic structure (28). However, in this study, gender was not found to be an independent risk factor, possibly because male patients are less likely to have a history of abdominal surgery compared to female patients, many of whom underwent cesarean sections prior to being diagnosed with colorectal cancer. A large case-control study including 47,574 patients with colorectal diseases demonstrated that smoking is associated with an increased risk of postoperative complications across all types of colorectal surgeries (29), our study failed to identify smoking as a risk factor for postoperative complications, which may be attributable to the effectiveness of our inpatient education program. Additionally, our research center places significant emphasis on the ERAS concept and prehabilitation strategies, routinely implementing measures such as oxygen supplementation and airway clearance during the perioperative period to optimize pulmonary function. The clinical experience of surgeons and the annual volume of surgeries they perform are also important factors influencing the incidence of postoperative complications, and this effect is often reflected in the duration of the operation.

Elderly patients with colorectal cancer frequently present with a higher prevalence of comorbidities, such as hypertension, diabetes mellitus, cardiovascular and cerebrovascular diseases, neurological disorders, and hepatic and renal insufficiency (30), elderly patients frequently exhibit physical vulnerabilities, including frailty, insufficient cardiopulmonary capacity, and preoperative nutritional deficiencies caused by prolonged chronic conditions related to chronic illnesses and malignancies. In the postoperative period, they are more susceptible to complications like heart failure and lung infections, and their ability to endure surgery is typically less than that of younger individuals (31, 32).

Albumin plays an essential role in preserving plasma colloid osmotic pressure, modulating immune reactions, supplying nutritional sustenance, enabling transport mechanisms, maintaining acid-base homeostasis, and demonstrating antioxidant properties (33). In the presence of hypoproteinemia (for instance, caused by liver or kidney disorders, or nutritional deficiencies), plasma colloid osmotic pressure decreases. This change facilitates fluid leakage from blood vessels, leading to tissue swelling or ascites formation. Consequently, the probability of postoperative complications, including wound separation and anastomotic failure, is heightened. Among colorectal cancer patients, preoperative malnutrition correlates with multiple unfavorable postoperative effects and a poorer long-term outlook. Individuals affected by malnutrition often exhibit significant weight reduction after surgery and demonstrate increased susceptibility to immune suppression, thereby raising the risk of infections and inflammatory reactions postoperatively (34–36).

The position of the tumor significantly affects the likelihood of postoperative complications.

The right colon enjoys a robust and redundant blood supply, primarily via the ileocolic (ICA), right colic (RCA), and middle colic arteries (MCA), which collectively form an extensive vascular arcade and ensure continuity of the marginal artery of Drummond. In contrast, the left colon relies predominantly on the inferior mesenteric artery (IMA); its terminal branches—the left colic and sigmoid arteries—exhibit physiologically vulnerable perfusion zones at the splenic flexure (Griffiths point) and the sigmoid–rectal junction (Sudeck point) (37). Furthermore, in approximately 30–40% of individuals, the collateral circulation between the superior and inferior mesenteric arteries is inadequate or absent, resulting in markedly reduced marginal artery perfusion pressure in the left colon relative to the right. Consequently, colonic–colonic or colonic–rectal anastomoses following left hemicolectomy lie within a hemodynamic watershed area, where tissue oxygen tension (PtO_2_) is 30–40% lower than in corresponding right-sided anastomoses (38). Additionally, the solid consistency of left colonic contents subjects the anastomosis to greater mechanical stress from formed stool, while also exposing it to high concentrations of digestive enzymes, bacterial endotoxins, and predominantly anaerobic microbiota—factors that directly impair mucosal integrity and predispose to fecal peritonitis. This leads to intense local inflammation, increased risk of intra-abdominal abscess formation, and suboptimal drainage due to anatomical constraints. By comparison, the right colon contains fluid luminal contents; thus, even in the event of anastomotic leakage, contamination is more readily dispersed and drained, markedly reducing the likelihood of localized septic complications (39, 40). From a surgical perspective, right-sided resections benefit from ample operative exposure, tension-free alignment of the ileum and transverse colon after mobilization, thin mesenteric fat, and clearly delineated vascular arcades. Left-sided anastomoses, however, are performed in the confined pelvic cavity, often under longitudinal tension between the proximal colon and rectal/sigmoid stump. In obese patients, thickened mesentery obscures vascular anatomy—particularly the marginal artery—increasing the risk of iatrogenic injury. Moreover, the thinner muscularis propria of the left colon compromises tissue resilience, resulting in inferior cutting–healing capacity compared with ileocolic anastomoses (41).The findings of this study further suggest that operative duration serves as an independent risk factor for the development of postoperative complications (42). Factors that may potentially lead to adverse outcomes due to prolonged surgery could include prolonged exposure to anesthetic agents, increased surgical stress reactions, and the continuation of pneumoperitoneum (43). Prior research suggests that extended periods under anesthesia are linked to a greater occurrence of postoperative nausea and vomiting, thromboembolic events, cardiopulmonary issues, infections at the surgical site, and higher mortality rates (44). The extended duration of surgery indirectly reflects its complexity. Variations in anatomical positioning or the concurrent resection of additional organs can lead to increased fatigue among the medical team, thereby heightening the risk of technical errors (45).

This research examines surgical outcomes in patients diagnosed with metastatic colorectal cancer (mCRC). Although earlier studies have primarily focused on long-term survival following post-treatment resection (PTR) surgery, this analysis emphasizes the occurrence of postoperative complications after PTR and constructs a reliable clinical prediction model. Initially, a nomogram is developed by incorporating an extensive range of risk factors, such as clinical and laboratory indicators, which enhances the accuracy of predicting complications. Subsequently, Lasso regression is utilized to select variables, ensuring that only the most significant predictors are included in the final model, thus reducing overfitting and improving its applicability across different contexts. Additionally, decision curve analysis (DCA) provides valuable insights into the practical utility of the nomogram, highlighting its capacity to support improved clinical decision-making processes. Finally, the robustness of the nomogram’s performance is assessed through ten-fold cross-validation and average calibration curve analysis, confirming its reliability across various datasets. While our study presents notable strengths, it also possesses limitations that require consideration. Primarily, being a single-center retrospective study might constrain the broader applicability of the findings. Furthermore, the limited sample size restricts the evaluation of the nomogram’s effectiveness to the current dataset, suggesting the need for external validation to ensure its generalizability. Various neoadjuvant treatment regimens exist, such as chemotherapy, immunotherapy, targeted therapy, and radiotherapy. The side effects associated with these regimens may differentially influence postoperative complications depending on the specific treatment modality (46, 47), stratified analysis of neoadjuvant therapy was not performed in this study owing to the limited sample size. In colorectal cancer patients with peritoneal metastasis, the combination of postoperative hyperthermic intraperitoneal chemotherapy (HIPEC) and cytoreductive surgery (CRS) has gained popularity as a treatment approach. However, certain studies have suggested an association between this treatment and the occurrence of anastomotic leakage. The primary aim of HIPEC is to eradicate free tumor cells and microscopic residual disease within the peritoneal cavity. Nevertheless, the exposure of abdominal organs to chemotherapeutic agents during HIPEC may potentially worsen intestinal obstruction and increase the risk of anastomotic leakage (39). According to another research report, the risk of anastomotic fistula following hyperthermic intraperitoneal chemotherapy (HIPEC) combined with cytoreductive surgery (CRS) is relatively low. When complete cytoreduction is achievable, the potential risk of anastomotic fistula should not deter the decision to proceed with resection (48). Finally, this study failed to account for potential confounding factors that could influence postoperative complications, such as preoperative psychological status (e.g., anxiety and depression), intraoperative adverse events, or postoperative management strategies. Future research should prioritize validating the nomogram in larger, multi-center cohorts to confirm its generalizability and robustness. Moreover, prospective studies are warranted to assess the nomogram’s impact on clinical decision-making and patient outcomes in real-world scenarios. Further investigations should also explore incorporating additional potential risk factors to improve the nomogram’s predictive accuracy.

## Conclusion

In conclusion, in cases where local or primary tumor resection is indicated, such interventions may confer a survival benefit in patients with metastatic colorectal cancer (49). this study determined four independent risk factors associated with moderate-to-severe complications in patients with metastatic colorectal cancer (mCRC) following primary tumor resection (PTR), and developed a nomogram model exhibiting outstanding predictive accuracy. This model can assist surgeons in designing more effective perioperative management strategies for high-risk patients. Future studies should prioritize external validation and further refinement of this model to maximize its clinical utility. 

## Data Availability

The original contributions presented in the study are included in the article/supplementary material. Further inquiries can be directed to the corresponding authors.

## References

[1] Morgan E Arnold M Gini A Lorenzoni V Cabasag CJ Laversanne M . Global burden of colorectal cancer in 2020 and 2040: incidence and mortality estimates from GLOBOCAN. Gut. (2023) 72:338–44. doi: 10.1136/gutjnl-2022-327736. PMID: 36604116

[2] Roshandel G Ghasemi-Kebria F Malekzadeh R . Colorectal cancer: epidemiology, risk factors, and prevention. Cancers (Basel). (2024) 16:1530. doi: 10.3390/cancers16081530. PMID: 38672612 PMC11049480

[3] Li J Li ZP Ruan WJ Wang W . Colorectal cancer screening: the value of early detection and modern challenges. World J Gastroenterol. (2024) 30:2726–30. doi: 10.3748/wjg.v30.i20.2726. PMID: 38855153 PMC11154673

[4] Kanth P Inadomi JM . Screening and prevention of colorectal cancer. BMJ. (2021) 374:n1855. doi: 10.1136/bmj.n1855. PMID: 34526356

[5] Chuang SC Huang CW Chen YT Ma CJ Tsai HL Chang TK . Effect of KRAS and NRAS mutations on the prognosis of patients with synchronous metastatic colorectal cancer presenting with liver-only and lung-only metastases. Oncol Lett. (2020) 20:2119–30. doi: 10.3892/ol.2020.11795. PMID: 32782529 PMC7400335

[6] Shin AE Giancotti FG Rustgi AK . Metastatic colorectal cancer: mechanisms and emerging therapeutics. Trends Pharmacol Sci. (2023) 44:222–36. doi: 10.1016/j.tips.2023.01.003. PMID: 36828759 PMC10365888

[7] Yang W Chen D Niu Y Wu G Huang Z Bi X . FOLFOXIRI plus cetuximab as conversion therapy for unresectable RAS/BRAF wild-type left-sided colorectal cancer with liver-limited metastases: a prospective dual-center pilot study. Front Oncol. (2024) 14:1375906. doi: 10.3389/fonc.2024.1375906. PMID: 38638850 PMC11024419

[8] Bolhuis K Kos M van Oijen MGH Swijnenburg RJ Punt CJA . Conversion strategies with chemotherapy plus targeted agents for colorectal cancer liver-only metastases: a systematic review. Eur J Cancer. (2020) 141:225–38. doi: 10.1016/j.ejca.2020.09.037. PMID: 33189037

[9] Tharin Z Blanc J Alaoui IC Bertaut A Ghiringhelli F . Influence of primary tumor location and resection on survival in metastatic colorectal cancer. World J Gastrointest Oncol. (2020) 12:1296–310. doi: 10.4251/wjgo.v12.i11.1296. PMID: 33250962 PMC7667454

[10] Kim JH Jin S Jeon MJ Jung HY Byun S Jung K . Survival benefit of palliative primary tumor resection based on tumor location in patients with metastatic colorectal cancer: a single-center retrospective study. Korean J Gastroenterol. (2020) 76:17–27. doi: 10.4166/kjg.2020.76.1.17. PMID: 32703916 PMC12319063

[11] Benavides M Gómez-España A García-Alfonso P González CG Viéitez JM Rivera F . Upfront primary tumour resection and survival in synchronous metastatic colorectal cancer according to primary tumour location and RAS status: pooled analysis of the Spanish Cooperative Group for the Treatment of Digestive Tumours (TTD). Eur J Surg Oncol. (2022) 48:1123–32. doi: 10.1016/j.ejso.2021.11.122. PMID: 34872775

[12] Oh NH Kim KJ . Outcomes and risk factors affecting mortality in patients who underwent colorectal emergency surgery. Ann Coloproctol. (2016) 32:133–8. doi: 10.3393/ac.2016.32.4.133. PMID: 27626023 PMC5019965

[13] Baer C Menon R Bastawrous S Bastawrous A . Emergency presentations of colorectal cancer. Surg Clin North Am. (2017) 97:529–45. doi: 10.1016/j.suc.2017.01.004. PMID: 28501245

[14] Jeong DS Kim YH Kim KJ . Surgical outcomes and risk factors in patients who underwent emergency colorectal surgery. Ann Coloproctol. (2017) 33:239–44. doi: 10.3393/ac.2017.33.6.239. PMID: 29354607 PMC5768479

[15] Sato T Watanabe M . The present status and developments of laparoscopic surgery for colorectal cancer. J Anus Rectum Colon. (2018) 1:1–6. doi: 10.23922/jarc.2016-010. PMID: 31583293 PMC6768680

[16] Durak D Alkurt EG Turhan VB Tutan B Sahiner IT Kendirci M . Comparison of short-term results of laparoscopic and open surgeries for colorectal cancer: a single-center experience. Cureus. (2022) 14:e24635. doi: 10.7759/cureus.24635. PMID: 35663698 PMC9152636

[17] Suda K Shimizu T Ishizuka M Miyashita S Niki M Shibuya N . Laparoscopic surgery reduced frequency of postoperative small bowel obstruction, and hospital stay compared with open surgery in a cohort of patients with colorectal cancer: a propensity score matching analysis [published correction appears in Surg Endosc. Surg Endosc. (2022) 36:8790–6. doi: 10.1007/s00464-022-09302-x. PMID: 35556165

[18] Ho MF Lai VC Ng DCK Ng SSM . Prognosis of patients with unresectable stage IV colon cancer undergoing primary tumor resection: a multicenter study of minimally symptomatic or asymptomatic primary tumor. Asian J Surg. (2023) 46:3710–5. doi: 10.1016/j.asjsur.2022.11.127. PMID: 36522225

[19] Tarantino I Warschkow R Worni M Cerny T Ulrich A Schmied BM . Prognostic relevance of palliative primary tumor removal in 37,793 metastatic colorectal cancer patients: a population-based, propensity score-adjusted trend analysis. Ann Surg. (2015) 262:112–20. doi: 10.1097/SLA.0000000000000860. PMID: 25373464

[20] Bolliger M Kroehnert JA Molineus F Kandioler D Schindl M Riss P . Experiences with the standardized classification of surgical complications (Clavien-Dindo) in general surgery patients. Eur Surg. (2018) 50:256–61. doi: 10.1007/s10353-018-0551-z. PMID: 30546385 PMC6267508

[21] Degerli MS Canturk AO Bozkurt H Alpay O Akinci M Altundal YE . Systematic assessment of complications after laparoscopic colorectal surgery for advanced colorectal cancer: a retrospective study using Clavien-Dindo classification, 5-year experience. Malawi Med J. (2022) 34:49–52. doi: 10.4314/mmj.v34i1.9. PMID: 37265825 PMC10230576

[22] Mukaka MM . Statistics corner: a guide to appropriate use of correlation coefficient in medical research. Malawi Med J. (2012) 24:69–71. 23638278 PMC3576830

[23] Lv J Liu YY Jia YT He JL Dai GY Guo P . A nomogram model for predicting prognosis of obstructive colorectal cancer. World J Surg Oncol. (2021) 19:337. doi: 10.1186/s12957-021-02445-6. PMID: 34857001 PMC8638364

[24] Xiang YC Liu XY Hai ZX Lv Q Zhang W Liu XR . Nomogram for predicting the development of pneumonia after colorectal cancer surgery. Sci Rep. (2025) 15:7417. doi: 10.1038/s41598-025-92106-5. PMID: 40033128 PMC11876627

[25] Ye C Wang X Sun Y Deng Y Huang Y Chi P . A nomogram predicting the difficulty of laparoscopic surgery for rectal cancer. Surg Today. (2021) 51:1835–42. doi: 10.1007/s00595-021-02338-x. PMID: 34296313

[26] Facciorusso A Di Maso M Serviddio G Vendemiale G Muscatiello N . Development and validation of a risk score for advanced colorectal adenoma recurrence after endoscopic resection. World J Gastroenterol. (2016) 22:6049–56. doi: 10.3748/wjg.v22.i26.6049. PMID: 27468196 PMC4948260

[27] Manekk RS Gharde P Gattani R Lamture Y . Surgical complications and its grading: a literature review. Cureus. (2022) 14:e24963. doi: 10.7759/cureus.24963. PMID: 35706751 PMC9187255

[28] Golse N Nunez J Mazzotta A Cano L Bergeat D Sulpice L . Personalized preoperative nomograms predicting postoperative risks after resection of perihilar cholangiocarcinoma. World J Surg. (2020) 44:3449–60. doi: 10.1007/s00268-020-05618-8. PMID: 32474628

[29] Sharma A Deeb AP Iannuzzi JC Rickles AS Monson JR Fleming FJ . Tobacco smoking and postoperative outcomes after colorectal surgery. Ann Surg. (2013) 258:296–300. doi: 10.1097/SLA.0b013e3182708cc5. PMID: 23059503

[30] Shi JX Cui J Li ZJ Ma FH Gao LL Cao XL . Analysis of clinical characteristics of elderly patients with colorectal cancer after surgery in different age groups Zhonghua Yi Xue Za Zhi. (2022) 102:563–8. doi: 10.3760/cma.j.cn112137-20211029-02399. PMID: 35196778

[31] Fagard K Casaer J Wolthuis A Flamaing J Milisen K Lobelle JP . Value of geriatric screening and assessment in predicting postoperative complications in patients older than 70 years undergoing surgery for colorectal cancer. J Geriatr Oncol. (2017) 8:320–7. doi: 10.1016/j.jgo.2017.07.008. PMID: 28781062

[32] Okabe H Ohsaki T Ogawa K Ozaki N Hayashi H Akahoshi S . Frailty predicts severe postoperative complications after elective colorectal surgery. Am J Surg. (2019) 217:677–81. doi: 10.1016/j.amjsurg.2018.07.009. PMID: 30473227

[33] Alajmi A Almehari A Alzahrani AR Aljurays Y Alzahrani N Aladel AM . Impact of preoperative serum albumin level on the outcome of colorectal cancer surgery. Cureus. (2024) 16:e57655. doi: 10.7759/cureus.57655. PMID: 38707022 PMC11070141

[34] Sofić A Rašić I Halilović E Mujić A Muslić D . Is preoperative hypoproteinemia associated with colorectal cancer stage and postoperative complications? Med Glas (Zenica). (2021) 18:450–5. doi: 10.17392/1353-21. PMID: 34190507

[35] Wang Y Wang H Jiang J Cao X Liu Q . Early decrease in postoperative serum albumin predicts severe complications in patients with colorectal cancer after curative laparoscopic surgery. World J Surg Oncol. (2018) 16:192. doi: 10.1186/s12957-018-1493-4. PMID: 30253767 PMC6156961

[36] Yang SP Wang TJ Huang CC Chang SC Liang SY Yu CH . Influence of albumin and physical activity on postoperative recovery in patients with colorectal cancer: an observational study. Eur J Oncol Nurs. (2021) 54:102027. doi: 10.1016/j.ejon.2021.102027. PMID: 34509088

[37] Veyrie N Ata T Muscari F Couchard AC Msika S Hay JM . Anastomotic leakage after elective right versus left colectomy for cancer: prevalence and independent risk factors. J Am Coll Surg. (2007) 205:785–93. doi: 10.1016/j.jamcollsurg.2007.06.284. PMID: 18035262

[38] Cirocchi R Randolph J Cheruiyot I Davies RJ Gioia S Henry BM . Discontinuity of marginal artery at splenic flexure and rectosigmoid junction: a systematic review and meta-analysis. Colorectal Dis. (2023) 25:1361–70. doi: 10.1111/codi.16624. PMID: 37317032

[39] Kneis B Wirtz S Weber K Denz A Gittler M Geppert C . Colon cancer microbiome landscaping: differences in right- and left-sided colon cancer and a tumor microbiome-ileal microbiome association. Int J Mol Sci. (2023) 24:3265. doi: 10.3390/ijms24043265. PMID: 36834671 PMC9963782

[40] Lianos GD Frountzas M Kyrochristou ID Sakarellos P Tatsis V Kyrochristou GD . What is the role of the gut microbiota in anastomotic leakage after colorectal resection? a scoping review of clinical and experimental studies. J Clin Med. (2024) 13:6634. doi: 10.3390/jcm13226634. PMID: 39597778 PMC11594793

[41] Matthiessen P Hallböök O Andersson M Rutegård J Sjödahl R . Risk factors for anastomotic leakage after anterior resection of the rectum. Colorectal Dis. (2004) 6:462–9. doi: 10.1111/j.1463-1318.2004.00657.x. PMID: 15521937

[42] Phan K Kim JS Kim JH Somani S Di’Capua J Dowdell JE . Anesthesia duration as an independent risk factor for early postoperative complications in adults undergoing elective ACDF. Glob Spine J. (2017) 7:727–34. doi: 10.1177/2192568217701105. PMID: 29238635 PMC5721997

[43] Unruh KR Bastawrous AL Kanneganti S Kaplan JA Moonka R Rashidi L . The impact of prolonged operative time associated with minimally invasive colorectal surgery: a report from the Surgical Care Outcomes Assessment Program. Dis Colon Rectum. (2024) 67:302–12. doi: 10.1097/DCR.0000000000002925. PMID: 37878484

[44] Ohkuma M Takano Y Goto K Okamoto A Koyama M Abe T . Significance of Naples prognostic score for postoperative complications after colorectal cancer surgery. Surg Today. 55, 1481–7 doi: 10.1007/s00595-025-03055-5. PMID: 40332592

[45] Jingtao Z Bin W Haoyu B Hexin L Xuejun Y Tinghao W . Prediction of postoperative complications following transanal total mesorectal excision in middle and low rectal cancer: development and internal validation of a clinical prediction model. Int J Colorectal Dis. (2024) 39:133. doi: 10.1007/s00384-024-04702-y. PMID: 39150559 PMC11329424

[46] Lordick F Gockel I . Chances, risks and limitations of neoadjuvant therapy in surgical oncology. Innov Surg Sci. (2016) 1:3–11. doi: 10.1515/iss-2016-0004. PMID: 31579713 PMC6753981

[47] Zhai JN Lei XK Wu AW . Regarding the selection of individualized therapy after neoadjuvant therapy for gastrointestinal tumors Zhonghua Wei Chang Wai Ke Za Zhi. (2024) 27:338–47. doi: 10.3760/cma.j.cn441530-20240227-00076. PMID: 38644238

[48] Herzberg J Acs M Guraya SY Schlitt HJ Honarpisheh H Strate T . Anastomotic leakage following cytoreductive surgery and hyperthermic intraperitoneal chemotherapy for colorectal cancer: a clinical cohort study. Surg Oncol. (2024) 54:102080. doi: 10.1016/j.suronc.2024.102080. PMID: 38663060

[49] Mazzotta AD Usdin N Samer D Tribillon E Gayet B Fuks D . Debulking hepatectomy for colorectal liver metastasis: analysis of risk factors for progression free survival. Surg Oncol. (2024) 55:102056. doi: 10.1016/j.suronc.2024.102056. PMID: 38531729

